# Antimicrobial Drug Prescription and *Neisseria gonorrhoeae* Susceptibility, United States, 2005–2013

**DOI:** 10.3201/eid2310.170488

**Published:** 2017-10

**Authors:** Robert D. Kirkcaldy, Monina G. Bartoces, Olusegun O. Soge, Stefan Riedel, Grace Kubin, Carlos Del Rio, John R. Papp, Edward W. Hook, Lauri A. Hicks

**Affiliations:** Centers for Disease Control and Prevention, Atlanta, Georgia, USA (R.D. Kirkcaldy, M.G. Bartoces, J.R. Papp, L.A. Hicks);; University of Washington, Seattle, Washington, USA (O.O. Soge);; Beth Israel Deaconess Medical Center, Boston, Massachusetts, USA (S. Riedel);; Texas Department of State Health Services, Austin, Texas, USA (G. Kubin);; Emory University, Atlanta (C. Del Rio);; University of Alabama at Birmingham, Birmingham, Alabama, USA (E.W. Hook III)

**Keywords:** gonorrhea, *Neisseria gonorrhoeae*, drug resistance, bacterial, antimicrobial drug, antibacterial agents, utilization, drug susceptibility, United States

## Abstract

We investigated whether outpatient antimicrobial drug prescribing is associated with *Neisseria gonorrhoeae* antimicrobial drug susceptibility in the United States. Using susceptibility data from the Gonococcal Isolate Surveillance Project during 2005–2013 and QuintilesIMS data on outpatient cephalosporin, macrolide, and fluoroquinolone prescribing, we constructed multivariable linear mixed models for each antimicrobial agent with 1-year lagged annual prescribing per 1,000 persons as the exposure and geometric mean MIC as the outcome of interest. Multivariable models did not demonstrate associations between antimicrobial drug prescribing and *N. gonorrhoeae* susceptibility for any of the studied antimicrobial drugs during 2005–2013. Elucidation of epidemiologic factors contributing to resistance, including further investigation of the potential role of antimicrobial drug use, is needed.

*Neisseria gonorrhoeae*, the causative pathogen of gonorrhea, has been designated an urgent antimicrobial drug resistance threat by the Centers for Disease Control and Prevention (CDC) (*1*). Since the introduction of antimicrobial drugs in the first half of the 20th century, *N. gonorrhoeae* has successively developed resistance to each antimicrobial agent recommended for gonorrhea treatment (*2*). In the United States, the prevalence of resistance in *N. gonorrhoeae* often varies by sex of partner and by geographic region (*3*,*4*). Prevalence is often greater in isolates from gay, bisexual, and other men who have sex with men (MSM) than those from men who have sex only with women (MSW), and prevalence is often highest in the West and lowest in the South (*4*). Resistant strains, in particular penicillinase-producing *N. gonorrhoeae *(PPNG), fluoroquinolone-resistant *N. gonorrhoeae*, and gonococcal strains with reduced cephalosporin susceptibility, seemed to emerge initially in the West (Hawaii and the West Coast) before spreading eastward across the country (*5*–*9*). These geographic patterns seem to support the idea that importation of resistant strains from other regions of the world, such as eastern Asia, is a primary factor of the emergence of resistant gonococci in the United States (*5*–*9*). Whereas antimicrobial drug prescribing patterns have been clearly associated with the emergence of resistance in other bacterial pathogens, the degree to which domestic antimicrobial use and subsequent selection pressure contributes to the emergence of gonococcal antimicrobial resistance in the United States is unclear (*10*–*13*). Using an ecologic approach, we sought to investigate the potential geographic and temporal association between antimicrobial drug susceptibility among US *N. gonorrhoeae* isolates and domestic outpatient antimicrobial drug prescribing rates in the United States during 2005–2013.

## Methods

### Data Sources

We used data from 3 sources: *N. gonorrhoeae* antimicrobial drug susceptibility data from the Gonococcal Isolate Surveillance Project (GISP), antimicrobial drug consumption data from IMS Health, and US Census data for population denominators. GISP is a CDC-supported sentinel surveillance system that has monitored gonococcal antimicrobial susceptibility in the United States since 1987 (*4*). GISP includes selected publicly funded sexually transmitted infection (STI) clinics in 25–30 cities and 4–5 regional laboratories each year. Each month, up to 25 *N. gonorrhoeae* urethral samples are collected consecutively from men with gonococcal urethritis attending participating STI clinics; these samples are submitted to regional laboratories for antimicrobial drug susceptibility testing according to a common protocol (*4*). Sampling men with gonococcal urethritis is an efficient means to detect gonococcal infections: urethral infections in men are likely to be symptomatic (prompting patients to seek healthcare), and gonococcal urethritis can be rapidly diagnosed by Gram stain (*4*). In addition, sampling of men allows for monitoring of susceptibility among infections in MSM and heterosexual sexual networks (*4*). 

We abstracted deidentified epidemiologic data from medical records. When analyzing data, we limited the number of isolates to <300/city/year to minimize overrepresentation of individual cities; we chose isolates at random for removal from the analytic dataset when >300 isolates were submitted from a site in a year. Gonococcal isolates collected at each clinic are subcultured at the clinic’s local public health laboratory on supplemented chocolate medium and frozen in trypticase soy broth containing 20% glycerol. Isolates are shipped monthly to 1 of the regional laboratories for β-lactamase production testing and agar dilution antimicrobial drug susceptibility testing. From 2005 through 2013, the testing panel consistently included azithromycin, ceftriaxone, ciprofloxacin, penicillin, spectinomycin, and tetracycline. Cefixime susceptibility testing was conducted from 2005 through 2006, temporarily halted in 2007 due to lack of availability of cefixime in the United States, and resumed in 2009. Cefpodoxime susceptibility testing was conducted from 2009 through 2012. Standardized bacterial suspensions are inoculated on Difco GC Medium Base supplemented with 1% IsoVitaleX Enrichment (Becton, Dickinson and Company Diagnostic Systems, Sparks, MD, USA).

For antimicrobial drug prescribing data, we extracted QuintilesIMS (Danbury, CT, USA) data on systemic oral antimicrobial drug prescriptions dispensed in the United States during 2005–2013. During the study period, QuintilesIMS captured >70% of all outpatient prescriptions in the United States, reconciled them to wholesale deliveries, and projected to 100% coverage of all prescription activity using a patented projection method based on a comprehensive sample of patient-deidentified prescription transactions (collected from pharmacies that report their entire pharmacy business to the company each week) (*14*). These data represent outpatient prescriptions across all payers and include county of prescriber (used in this analysis); data are from community pharmacies and federal government and nongovernmental mail service pharmacies. The IMS projection method standardizes these data into estimated prescription counts and uses geospatial methods to align the estimated prescriptions for the non-sample pharmacies to prescribers with observed prescribing behaviors for the same product in nearby sample pharmacies. The method is routinely validated at various levels of granularity by IMS Health statistical and analytic teams (*15*). We obtained population data on age, sex, and county from US Census bridged-race population estimates published by the CDC. 

### Data Analysis

We restricted the analysis to GISP sites that participated continuously from 2005 through 2013. We focused on azithromycin, cefixime, ceftriaxone, and ciprofloxacin because these agents are currently recommended for gonorrhea treatment (azithromycin and ceftriaxone), are in the same antimicrobial class as a recommended antimicrobial drug (cefixime), or are widely used for outpatient treatment of non-STD infections or are of renewed interest because of potential novel diagnostics for detection of resistance determinants (ciprofloxacin) (*16*,*17*). We calculated geometric mean MICs and the percentage of isolates with resistance or reduced susceptibility for each antimicrobial drug by GISP site and year. Per Clinical and Laboratory Standards Institute criteria, we categorized ciprofloxacin resistance as MIC >1 µg/mL (*18*). In the absence of established resistance breakpoints for other antimicrobial drugs of interest, we categorized reduced cefixime susceptibility as >0.25 µg/mL, reduced ceftriaxone susceptibility as >0.125 µg/mL, and reduced azithromycin susceptibility as >2.0 µg/mL.

Using IMS data for each county corresponding to each of the 23 included GISP sites, we summarized antimicrobial drug prescription counts by specific antimicrobial drug (azithromycin, cefixime, ceftriaxone, and ciprofloxacin) and aggregated antimicrobial category (macrolides, cephalosporins, and fluoroquinolones). To reflect the sex and age distribution of men with gonorrhea sampled in GISP, we limited the antimicrobial drug prescription counts to prescriptions to men 10–59 years of age. We calculated prescription rates for the aggregated antimicrobial drug categories (defined as the number of prescriptions per 1,000 men 10–59 years of age) by county and year using US Census data for denominators. Using the prescribing rate for each antimicrobial category by county and year, we calculated the median prescribing rate for each county across years. The degree of overlap between the county and catchment area of the corresponding STD clinic is expected to be somewhat imprecise and vary by geographic site: some clinic catchment areas may include a small section of a large county, whereas other catchment areas may extend beyond the borders of the corresponding county.

We performed linear regression analyses in which the dependent variable was antimicrobial susceptibility (geometric mean MIC) at each GISP site and the independent variable of interest was the prior year prescription rate (i.e., the prescribing rate during the year before the year corresponding to the antimicrobial drug susceptibility results) at each county and year. We considered 2 representations of the prescribing rate variable: the original lag variable and the centered lag variable (*19*,*20*). We used the noncentered lag variable to calculate the results. We performed separate longitudinal models for each drug by geometric mean MIC and the rate of prescribing of the corresponding antimicrobial class (azithromycin susceptibility and macrolide prescribing; cefixime and ceftriaxone susceptibility and cephalosporin prescribing; and ciprofloxacin susceptibility and fluoroquinolone prescribing) (*19*,*20*). We performed exploratory analyses to determine if there was a linear relationship between the susceptibility outcome and time. The linear assumption was satisfied, so we did not perform any transformation. To examine the association between susceptibility outcomes and prescribing rate, we then constructed multivariable linear mixed models for repeated measures with intercept and time as random effects. The models included 3 potential confounders based on a priori decisions (as these variables have been found to be associated with antimicrobial drug prescribing and/or gonococcal susceptibility): geographic region; sex of sex partner (defined as the percentage of MSM at each GISP site per year, based on GISP data); and race (defined as the percentage of men with urethral gonorrhea who were black or African American at each GISP site per year, based on GISP data) (*3*,*4*,*15*,*21*). We conducted all analyses in SAS version 9.3 (SAS Institute, Inc., Cary, NC, USA), using Proc Mixed for restricted maximum-likelihood estimation for small size samples. We calculated CIs at α = 0.05 to determine statistically significant associations.

## Results

### Antimicrobial Susceptibility

Of 33 GISP sites that participated at some point during 2005 through 2013, 23 participated continuously and were included. From these sites, 44,957 isolates were collected and submitted to GISP (range per site in a given year 49–393) and, after removal of observations if >300 isolates were submitted by a site in a year, we included data from 43,852 (97.5%) isolates in the analysis. The percentage of gonococcal isolates with reduced cefixime susceptibility increased from 0.1% in 2005 to 1.6% in 2011 and decreased to 0.5% by 2013 (Table 1). Overall, geometric mean cefixime MICs increased slightly from 2006 to 2009 and then remained stable. The percentage of isolates with reduced ceftriaxone susceptibility increased slightly from 2005 to 2011 and then decreased; the geometric mean increased slightly from 2006 to 2007 and then remained stable. The percentage of isolates with reduced azithromycin susceptibility varied between 0.2% and 0.6%; the geometric mean appeared to peak in 2008 and increased again during 2011–2013. The percentage of isolates with ciprofloxacin resistance increased during 2005–2007 and increased again during 2009–2013; the geometric mean MIC increased during 2005–2013. For each antimicrobial drug, geometric mean MICs varied by site and year (Technical Appendix Tables 1–4, 8). Sites with the highest median cefixime and ciprofloxacin geometric mean MICs were in the West; those with the lowest were in the South and Midwest (Technical Appendix Tables 1, 4). Sites with highest median azithromycin geometric means were in the Midwest and West, and those with the lowest were in the South (Technical Appendix Table 3). We found little variation in median ceftriaxone geometric mean MICs across sites (Technical Appendix 2).

**Table 1:** Antimicrobial drug resistance and reduced susceptibility in gonococcal isolates by drug, Gonococcal Isolate Surveillance Project, United States, 2005–2013*

Results†	Cefixime	Ceftriaxone	Azithromycin	Ciprofloxacin
2005				
Geometric mean MIC	0.009	0.006	0.189	0.011
Reduced susceptibility, %	0.1	0.1	0.6	10.1
2006				
Geometric mean MIC	0.010	0.005	0.204	0.016
Reduced susceptibility, %	0.1	0.1	0.3	15.4
2007				
Geometric mean MIC	NT	0.010	0.240	0.027
Reduced susceptibility, %	–	0.1	0.5	16.0
2008				
Geometric mean MIC	NT	0.010	0.242	0.024
Reduced susceptibility, %	–	0.1	0.2	14.7
2009				
Geometric mean MIC	0.020	0.010	0.192	0.031
Reduced susceptibility, %	0.9	0.3	0.3	10.8
2010				
Geometric mean MIC	0.020	0.010	0.174	0.039
Reduced susceptibility, %	1.6	0.4	0.6	14.2
2011				
Geometric mean MIC	0.020	0.010	0.171	0.039
Reduced susceptibility, %	1.6	0.4	0.3	14.4
2012				
Geometric mean MIC	0.020	0.010	0.183	0.042
Reduced susceptibility, %	1.0	0.3	0.3	16.1
2013				
Geometric mean MIC	0.021	0.010	0.202	0.043
Reduced susceptibility, %	0.5	0.1	0.6	17.1

*Results are for 23 sites that participated in GISP for the entire study period. Cefixime MIC testing range was 0.001–0.5 µg/mL during 2005–2006 and 0.015–0.5 µg/mL during 2009–2013; ceftriaxone MIC testing range was 0.001–2.0 µg/mL during 2005–2006 and 0.008–2.0 µg/mL during 2007–2013; azithromycin MIC testing range was 0.008–16 µg/mL during 2005–2006 and 0.03–16 µg/mL during 2007–2013; ciprofloxacin MIC testing range was 0.001–16 µg/mL during 2005–2006 and 0.008–16 µg/mL during 2007–2013. NT, not tested. †Reduced susceptibility indicates isolate’s resistance or reduced susceptibility to the indicated drug. Reduced cefixime susceptibility was defined as MIC ≥0.25 µg/ml, reduced ceftriaxone susceptibility MIC ≥0.125 µg/mL, reduced azithromycin susceptibility MIC ≥2 µg/mL, and ciprofloxacin resistance defined as MIC ≥1 µg/mL.

### Antimicrobial Drug Use

Counties with the highest cephalosporin, macrolide, and fluoroquinolone prescribing rates, such as Jefferson County, Alabama, and Oklahoma County, Oklahoma, were located in the South (Technical Appendix Tables 5–7). Counties with the lowest prescribing rates, such as Multnomah County, Oregon, and San Diego and San Francisco, California, were located in the West. Cephalosporin prescribing rates increased in many counties but decreased in sites such as those in Honolulu, Hawaii, and Los Angeles, California (Technical Appendix Table 5). During 2005–2013, macrolide prescribing increased in all counties (Technical Appendix Table 6). Fluoroquinolone prescribing increased in most counties, with the largest absolute increases occurring in counties in the South (Technical Appendix Table 7). The multivariable models did not demonstrate associations between *N. gonorrhoeae* susceptibility and antimicrobial drug prescribing for any of the studied antimicrobial drugs (Table 2).

**Table 2:** Adjusted linear regression coefficients for change in antimicrobial geometric mean MIC associated with 10% increase in corresponding antimicrobial prescribing rate for 23 sites, Gonococcal Isolate Surveillance Project, United States, 2005–2013*

Effect	β coefficient	SE	d.f.	95% CI of β coefficient
Azithromycin				
Time	−0.0087	0.003	155	−0.0146, −0.0029
Macrolide prescribing†	−0.0155	0.002	155	−0.0502, 0.0191
Cefixime				
Time	0.0011	0.0001	109	0.0008, 0.0014
Cephalosporin prescribing‡	0.0016	0.0013	109	−0.0010, 0.0041
Ceftriaxone				
Time	0.0004	0.0001	155	0.0002, 0.0005
Cephalosporin prescribing‡	0.0002	0.0006	155	−0.0009, 0.0013
Ciprofloxacin				
Time	0.0004	0.0021	155	−0.0038, 0.0045
Fluoroquinolone prescribing§	0.0004	0.0230	155	−0.0451, 0.0458

*All models were adjusted for percent of MSM at each site (using GISP data), race (percentage of men coded as black versus non-black in GISP data) percentage,and geographic region. Time was based on 1-year intervals. Estimate is statistically significant if the 95% CI of β coefficient does not cross 0. †Per 10% increase in macrolide prescribing during the previous year; includes azithromycin, clarithromycin, and erythromycin. ‡Per 10% increase in cephalosporin prescribing during the previous year; includes cefaclor, cefadroxil, cefdinir, cefditoren pivoxil, cefixime, cefpodoxime proxetil, cefprozil, ceftibuten, cefuroxime axetil, cephalexin, cephradine, and loracarbef. §Per 10% increase in fluoroquinolone prescribing during the previous year; includes ciprofloxacin, gemifloxacin, levofloxacin, moxifloxacin, norfloxacin, ofloxacin, and trovafloxacin.

## Discussion

Using an ecologic approach, we did not find an association between population-level outpatient prescribing rates of clinically relevant antimicrobial drugs and *N. gonorrhoeae* antimicrobial drug susceptibility among urethral isolates from men in the United States. Prescribing rates were lowest in sites both where ciprofloxacin resistance and reduced cefixime susceptibility initially emerged and where the prevalence of resistance or reduced susceptibility has been highest, such as Honolulu, Hawaii, and West Coast sites (*4*,*22*,*23*). Conversely, prescribing rates are highest in the southern United States, the region where the prevalence of gonococcal resistance has tended to be the lowest (*4*).

Bacterial antimicrobial drug resistance is clearly broadly linked to antimicrobial drug use, but the association is probably complex, interacting through several possible mechanisms and varying by bacteria, mode of transmission, antimicrobial drug, prevalence of resistance, and geographic location (*24*). For some bacterial pathogens, such as *Streptococcus pneumoniae* and *Escherchia coli,* associations between population-level antimicrobial drug prescribing and resistance in the United States and Europe have been described (*10*–*13*). We did not find such an association for *N. gonorrhoeae*. 

There are at least 2 possible explanations for the apparent lack of county-level association between domestic antimicrobial drug prescribing and *N. gonorrhoeae* susceptibility. First, factors other than population-level prescribing rates, such as importation of resistant strains from other countries, might contribute to emergence of gonococcal resistance in the United States. Previously published epidemiologic data have strongly suggested that resistant strains, such as PPNG and fluoroquinolone-resistant *N. gonorrhoeae*, and strains with reduced cefixime susceptibility (also investigated with genomic data), emerged initially in other parts of the world, particularly eastern Asia, and subsequently spread to the United States through Hawaii and the West Coast (*5*–*9*).

Second, it is possible that our data sources, methodology, or both lacked sufficient sensitivity to detect an association. The time period analyzed might have played a role: antimicrobial drug prescribing could conceivably be of greater or lesser importance during different phases of the emergence or persistence of resistance. As an inherent limitation of an ecologic approach, it is also possible that the prescribing rates we used in our analysis do not necessarily reflect antimicrobial drug use patterns among persons at risk for gonorrhea. For example, men seeking care in publicly funded STI clinics may be underinsured and thus lack access to routine medical care and be less likely to receive antimicrobial drug prescriptions. On the other hand, men diagnosed with gonorrhea in STI clinics may have been exposed to repeated antimicrobial drug courses for repeated STIs. Antimicrobial drug use might differ between MSM and MSW, so the availability of population-level drug prescribing data that include the sex of sex partners would be helpful.

Future investigations of associations between antimicrobial drug use and gonococcal susceptibility may also be strengthened by the availability of the indication for treatment (not available in the QuintilesIMS data), allowing for analyses linking susceptibility to antimicrobial drug use specifically for gonorrhea treatment. Investigators have previously identified links between individual-level antimicrobial drug use and gonococcal resistance in the United States (*25*,*26*). Nearly 30 years ago, Zenilman et al. found that persons with gonorrhea in Dade County, Florida, who had medicated themselves with illicit antibacterial drugs were more likely to be infected with PPNG than penicillin-sensitive strains (odds ratio 3.6; 95% CI 1.9–6.8) (*25*). Of note, our dataset does not include illicit or nonprescribed antimicrobial drugs. Among persons with gonorrhea in California during 2000–2003, antimicrobial drug use in the 3 months before diagnosis with gonorrhea was independently associated with infection with a fluoroquinolone-resistant strain (*26*). Using multisite GISP data for 2005–2010, we previously found that recent antimicrobial drug use was independently associated with *N. gonorrhoeae* ciprofloxacin, penicillin, and tetracycline resistance among men with gonococcal urethritis (*3*). However, the magnitude of the association between resistance and antimicrobial drug use was dwarfed by the magnitude of the association between resistance and geographic region and sex of sex partner, and use of an antimicrobial drug was not associated with reduced susceptibility to azithromycin, cefixime, and ceftriaxone.

The antimicrobial drugs that clinicians choose to treat gonorrhea may influence the susceptibility of *N. gonorrhoeae* populations. In the early 1970s, an increase in the recommended dosage of penicillin in response to increased resistance was followed by a plateau in penicillin resistance; experts speculated that the updated and highly effective treatment schedule retarded the selection of resistant mutants (*27*). Recently, cephalosporin susceptibility in the United States appeared to improve following updates in CDC treatment guidelines that recommended routine dual therapy, a preference for injectable ceftriaxone over oral cefixime, and a higher ceftriaxone dose (*28*). In contrast, some gonorrhea treatment approaches might promote resistance. Treatment with azithromycin alone is not recommended because of concerns about the ease with which *N. gonorrhoeae* can develop macrolide drug resistance; previously published cases seem to illustrate selection of higher azithromycin MICs following gonorrhea treatment with azithromycin monotherapy (*29*–*31*). Spectinomycin resistance was observed to emerge rapidly among US service members stationed in South Korea after spectinomycin was adopted as the primary gonorrhea treatment by the US military (following the emergence of PPNG) (*32*).

Emergence and persistence of gonococcal-resistant phenotypes is probably influenced by a complex (and not yet fully understood) interplay of bacterial and host factors, such as the ease with which the gonococcal strain can acquire necessary mutation(s); the effect of the mutation(s) on bacterial fitness; the anatomic site of infection (which can influence symptomatology, likelihood of treatment success, and coexistence of *N. gonorrhoeae* with other bacteria with which DNA may be shared); host mobility (including international travel); host sexual behavior; the nature of the sexual network within which the resistant strain emerges; prevalence of resistance; provider screening practices; and antimicrobial drug exposure (*32*–*37*). Furthermore, the relative importance of each factor may differ by resistance phenotype. The framework posited by Lipsitch and Samore may prove useful for considering mechanisms by which antimicrobial drugs might contribute to *N. gonorrhoeae* resistance, such as emergence of resistance during treatment or clearance of a susceptible majority bacterial population and subsequent transmission of a resistant minority population (*24*,*29*). However, much work remains to be done to understand these complex relationships.

Our analysis has other limitations. Ecologic analyses are limited by the potential for unmeasured and uncontrolled confounding. Conclusions of this ecologic analysis are based on counties or geographic site, rather than individual patients. Prescribing data were derived from counties that in some instances do not fully overlap with the STD clinic catchment areas from which the susceptibility data were derived. Our analyses were limited to data from men. However, the inclusion of data from women is unlikely to have influenced the results: women may consume more antimicrobial drugs than men, and gonococcal isolates from women tend to be more susceptible to antimicrobial drugs than those from men (similar to isolates from MSW and substantially more susceptible than isolates from MSM) (*38*,*39*). An important caveat is that our findings are only applicable to the United States: they should not be extrapolated to other countries and regions. It is possible that rates of population-level antimicrobial drug prescribing or use in other countries may select for resistant gonococcal strains, which in turn may spread across international borders. Further investigation to understand region- or county-specific factors contributing to resistance is urgently needed.

The findings of our analysis suggest that population-wide domestic antimicrobial drug prescribing rates might not play a prominent role in the emergence of gonococcal resistance in the United States. Other means, such as importation from other countries, might play larger roles. Through this lens, enhanced surveillance for and public health capacity to respond to imported resistant strains are important strategies. However, it is possible that the choice of antimicrobial drugs that clinicians prescribe for gonorrhea therapy might influence the persistence or spread of resistant gonococcal strains that emerge in the United States. US-based healthcare providers should treat gonorrhea according to CDC STI treatment guidelines with dual therapy of 250 mg ceftriaxone as a single intramuscular dose plus 1 g azithromycin orally (*16*). The remarkable ability of *N. gonorrhoeae* to develop resistance to each antimicrobial drug used for treatment (*2*), combined with the declining number of new drugs (*40*), highlight the need to develop and apply interventions to slow the emergence and spread of gonococcal resistance.

Technical Appendix Additional information from the Gonococcal Isolate Surveillance Project, United States, 2005–2013.
