# Supplementary material for: Antimicrobial Drug Prescription and Neisseria gonorrhoeae Susceptibility, United States, 2005–2013
Source: Emerg Infect Dis. 2017 Oct;23(10):1657–63. doi: 10.3201/eid2310.170488 (PMC5621530; doi:10.3201/eid2310.170488)
Supplement: Technical Appendix — Additional information from the Gonococcal Isolate Surveillance Project, United States, 2005–2013. [file 17-0488-Techapp-s1.pdf]

Article DOI: https://doi.org/10.3201/eid2310.170488

Antimicrobial Drug Prescription and *Neisseria gonorrhoeae* Susceptibility, United States, 2005–2013

Technical Appendix

Technical Appendix Table 1. Geometric mean of cefixime MICs by site and year, Gonococcal Isolate Surveillance Project (GISP), 2005–2013*

GISP site	Corresponding county in analysis	2005	2006	2007, 2008	2009	2010	2011	2012	2013
Northeast									
Philadelphia, Pennsylvania	Philadelphia	0.016	0.009	–	0.020	0.019	0.021	0.021	0.020
Midwest									
Chicago, Illinois	Cook County	0.007	0.007	–	0.021	0.019	0.02	0.019	0.019
Cleveland, Ohio	Cuyahoga County	0.007	0.008	–	0.017	0.017	0.017	0.018	0.017
Minneapolis, Minnesota	Hennepin County	0.007	0.009	–	0.024	0.023	0.019	0.022	0.022
South									
Baltimore, Maryland	Baltimore County	0.014	0.008	–	0.019	0.022	0.023	0.023	0.019
Dallas, Texas	Dallas County	0.005	0.007	–	0.018	0.019	0.019	0.019	0.019
Atlanta, Georgia	Fulton County	0.011	0.011	–	0.016	0.016	0.018	0.017	0.016
Greensboro, North Carolina	Guilford County	0.009	0.007	–	0.016	0.017	0.018	0.019	0.016
Birmingham, Alabama	Jefferson County	0.013	0.007	–	0.019	0.018	0.019	0.019	0.019
Miami, Florida	Miami-Dade County	0.014	0.011	–	0.017	0.018	0.019	0.019	0.018
Oklahoma City, Oklahoma	Oklahoma County	0.013	0.008	–	0.017	0.018	0.019	0.020	0.017
New Orleans, Louisiana	Orleans Parish	0.012	0.011	–	0.017	0.018	0.018	0.017	0.017
West									
Albuquerque, New Mexico	Bernalillo County	0.012	0.013	–	0.018	0.018	0.02	0.019	0.018
Las Vegas, Nevada	Clark County	0.006	0.008	–	0.021	0.020	0.018	0.020	0.019
Denver, Colorado	Denver County	0.010	0.009	–	0.020	0.019	0.020	0.021	0.020
Honolulu, Hawaii	Honolulu County	0.005	0.010	–	0.026	0.028	0.020	0.018	0.020
Seattle, Washington	King County	0.006	0.010	–	0.030	0.030	0.030	0.030	0.030
Los Angeles, California	Los Angeles County	0.011	0.014	–	0.026	0.025	0.023	0.022	0.022
Phoenix, Arizona	Maricopa County	0.012	0.014	–	0.022	0.020	0.018	0.019	0.019
Portland, Oregon	Multnomah County	0.009	0.011	–	0.024	0.023	0.022	0.022	0.022
Orange County, California	Orange County	0.014	0.015	–	0.023	0.026	0.024	0.030	0.024
San Diego, California	San Diego County	0.015	0.017	–	0.028	0.030	0.029	0.028	0.028
San Francisco, California	San Francisco County	0.007	0.012	–	0.023	0.023	0.023	0.023	0.023

*Cefixime susceptibility not tested in 2007 and 2008. MIC range was 0.001–0.5 µg/mL in 2005–2006; range was 0.015–0.5 µg/mL during 2009–2013.

Technical Appendix Table 2. Geometric mean ceftriaxone MICs by GISP site and year, Gonococcal Isolate Surveillance Project (GISP), 2005–2013*

GISP site	Corresponding county in analysis	2005	2006	2007	2008	2009	2010	2011	2012	2013
Northeast										
Philadelphia, Pennsylvania	Philadelphia	0.008	0.006	0.011	0.010	0.011	0.011	0.009	0.010	0.009
Midwest										
Chicago, Illinois	Cook County	0.004	0.004	0.010	0.010	0.011	0.011	0.010	0.010	0.009
Cleveland, Ohio	Cuyahoga County	0.003	0.004	0.009	0.009	0.009	0.009	0.009	0.009	0.009
Minneapolis, Minnesota	Hennepin County	0.004	0.005	0.011	0.012	0.014	0.012	0.012	0.010	0.012
South										
Baltimore, Maryland	Baltimore County	0.006	0.004	0.009	0.009	0.009	0.010	0.009	0.010	0.009
Dallas, Texas	Dallas County	0.005	0.004	0.009	0.009	0.009	0.009	0.009	0.010	0.0100
Atlanta, Georgia	Fulton County	0.005	0.005	0.010	0.010	0.009	0.009	0.009	0.010	0.010
Greensboro, North Carolina	Guilford County	0.004	0.004	0.009	0.009	0.010	0.009	0.009	0.010	0.008
Birmingham, Alabama	Jefferson County	0.005	0.003	0.009	0.009	0.009	0.009	0.009	0.009	0.008
Miami, Florida	Miami-Dade County	0.006	0.006	0.010	0.009	0.010	0.10	0.010	0.011	0.012
Oklahoma City, Oklahoma	Oklahoma County	0.005	0.004	0.009	0.009	0.009	0.008	0.009	0.010	0.010
New Orleans, Louisiana	Orleans Parish	0.006	0.006	0.010	0.009	0.010	0.009	0.010	0.009	0.009
West										
Albuquerque, New Mexico	Bernalillo County	0.006	0.005	0.011	0.009	0.011	0.010	0.011	0.011	0.010
Las Vegas, Nevada	Clark County	0.004	0.005	0.012	0.011	0.010	0.010	0.010	0.009	0.010
Denver, Colorado	Denver County	0.005	0.004	0.010	0.009	0.009	0.010	0.010	0.011	0.011
Honolulu, Hawaii	Honolulu County	0.005	0.005	0.010	0.010	0.011	0.011	0.013	0.011	0.010
Seattle, Washington	King County	0.006	0.006	0.011	0.010	0.011	0.013	0.013	0.014	0.016
Los Angeles, California	Los Angeles County	0.006	0.007	0.010	0.009	0.010	0.011	0.012	0.013	0.013
Phoenix, Arizona	Maricopa County	0.006	0.006	0.010	0.011	0.011	0.011	0.010	0.009	0.009
Portland, Oregon	Multnomah County	0.008	0.005	0.010	0.012	0.010	0.013	0.015	0.013	0.013
Orange County, California	Orange County	0.008	0.008	0.011	0.012	0.012	0.012	0.011	0.011	0.012
San Diego, California	San Diego County	0.008	0.009	0.013	0.012	0.011	0.012	0.013	0.012	0.011
San Francisco, California	San Francisco County	0.008	0.007	0.010	0.010	0.009	0.010	0.012	0.012	0.013

* MIC range was 0.001–2.0 µg/mL in 2005–2006; range was 0.008–2.0 µg/mL during 2007–2013.

Technical Appendix Table 3. Geometric mean azithromycin MICs by GISP site and year, Gonococcal Isolate Surveillance Project (GISP), 2005–2013*

GISP site	Corresponding county in analysis	2005	2006	2007	2008	2009	2010	2011	2012	2013
Northeast										
Philadelphia, Pennsylvania	Philadelphia	0.31	0.273	0.369	0.343	0.328	0.298	0.228	0.226	0.24
Midwest										
Chicago, Illinois	Cook County	0.226	0.231	0.291	0.267	0.244	0.324	0.269	0.276	0.285
Cleveland, Ohio	Cuyahoga County	0.197	0.258	0.278	0.265	0.212	0.294	0.303	0.213	0.229
Minneapolis, Minnesota	Hennepin County	0.242	0.28	0.292	0.361	0.314	0.347	0.361	0.284	0.385
South										
Baltimore, Maryland	Baltimore County	0.264	0.261	0.316	0.251	0.219	0.234	0.191	0.211	0.211
Dallas, Texas	Dallas County	0.1	0.081	0.096	0.102	0.106	0.068	0.084	0.137	0.162
Atlanta, Georgia	Fulton County	0.181	0.188	0.167	0.297	0.079	0.097	0.029	0.099	0.118
Greensboro, North Carolina	Guilford County	0.128	0.194	0.194	0.256	0.15	0.119	0.1	0.115	0.175
Birmingham, Alabama	Jefferson County	0.248	0.289	0.324	0.325	0.207	0.228	0.157	0.171	0.166
Miami, Florida	Miami-Dade County	0.166	0.178	0.192	0.245	0.199	0.109	0.104	0.1000	0.153
Oklahoma City, Oklahoma	Oklahoma County	0.222	0.257	0.271	0.242	0.229	0.183	0.153	0.143	0.219
New Orleans, Louisiana	Orleans Parish	0.162	0.239	0.184	0.234	0.103	0.125	0.096	0.112	0.139
West										
Albuquerque, New Mexico	Bernalillo County	0.182	0.182	0.258	0.239	0.114	0.123	0.09	0.136	0.113
Las Vegas, Nevada	Clark County	0.216	0.259	0.294	0.276	0.243	0.317	0.299	0.192	0.23
Denver, Colorado	Denver County	0.18	0.161	0.223	0.128	0.101	0.065	0.13	0.183	0.161
Honolulu, Hawaii	Honolulu County	0.100	0.134	0.168	0.206	0.187	0.148	0.152	0.21	0.133
Seattle, Washington	King County	0.142	0.156	0.191	0.154	0.162	0.158	0.147	0.213	0.234
Los Angeles, California	Los Angeles County	0.195	0.204	0.247	0.189	0.181	0.127	0.145	0.213	0.22
Phoenix, Arizona	Maricopa County	0.193	0.182	0.256	0.295	0.316	0.383	0.353	0.232	0.300
Portland, Oregon	Multnomah County	0.149	0.155	0.200	0.235	0.259	0.157	0.219	0.238	0.217
Orange County, California	Orange County	0.253	0.280	0.273	0.406	0.508	0.496	0.287	0.218	0.279
San Diego, California	San Diego County	0.210	0.233	0.315	0.424	0.438	0.460	0.292	0.271	0.236
San Francisco, California	San Francisco County	0.137	0.188	0.210	0.216	0.204	0.126	0.150	0.244	0.238

* MIC testing range was 0.008–16 µg/mL in 2005–2006; range was 0.03–16 µg/mL during 2007–2013.

Technical Appendix Table 4. Geometric mean ciprofloxacin MICs by GISP site and year, Gonococcal Isolate Surveillance Project (GISP), 2005–2013*

GISP site	Corresponding county in analysis	2005	2006	2007	2008	2009	2010	2011	2012	2013
Northeast										
Philadelphia, Pennsylvania	Philadelphia	0.015	0.036	0.063	0.033	0.042	0.038	0.025	0.053	0.035
Midwest										
Chicago, Illinois	Cook County	0.009	0.007	0.016	0.013	0.029	0.051	0.029	0.026	0.021
Cleveland, Ohio	Cuyahoga County	0.008	0.007	0.010	0.011	0.016	0.016	0.016	0.016	0.017
Minneapolis, Minnesota	Hennepin County	0.009	0.008	0.017	0.014	0.076	0.059	0.063	0.03	0.042
South										
Baltimore, Maryland	Baltimore County	0.005	0.004	0.009	0.012	0.02	0.028	0.016	0.021	0.05
Dallas, Texas	Dallas County	0.006	0.006	0.014	0.013	0.024	0.028	0.022	0.032	0.029
Atlanta, Georgia	Fulton County	0.007	0.008	0.01	0.018	0.021	0.02	0.024	0.024	0.031
Greensboro, North Carolina	Guilford County	0.007	0.005	0.012	0.011	0.017	0.017	0.02	0.023	0.017
Birmingham, Alabama	Jefferson County	0.005	0.004	0.015	0.014	0.029	0.032	0.056	0.036	0.029
Miami, Florida	Miami-Dade County	0.011	0.024	0.035	0.025	0.033	0.041	0.064	0.062	0.062
Oklahoma City, Oklahoma	Oklahoma County	0.005	0.005	0.012	0.011	0.018	0.015	0.017	0.019	0.022
New Orleans, Louisiana	Orleans Parish	0.010	0.011	0.029	0.023	0.025	0.034	0.033	0.029	0.036
West										
Albuquerque, New Mexico	Bernalillo County	0.009	0.011	0.026	0.014	0.024	0.066	0.064	0.048	0.042
Las Vegas, Nevada	Clark County	0.009	0.009	0.032	0.029	0.032	0.024	0.026	0.052	0.074
Denver, Colorado	Denver County	0.013	0.017	0.025	0.018	0.033	0.042	0.049	0.044	0.047
Honolulu, Hawaii	Honolulu County	0.020	0.076	0.075	0.137	0.271	0.128	0.096	0.042	0.034
Seattle, Washington	King County	0.012	0.099	0.129	0.123	0.073	0.500	0.214	0.119	0.262
Los Angeles, California	Los Angeles County	0.018	0.032	0.041	0.027	0.036	0.048	0.084	0.122	0.12
Phoenix, Arizona	Maricopa County	0.010	0.016	0.015	0.026	0.039	0.039	0.028	0.029	0.030
Portland, Oregon	Multnomah County	0.029	0.046	0.070	0.429	0.059	0.093	0.075	0.06	0.039
Orange County, California	Orange County	0.045	0.073	0.129	0.081	0.048	0.061	0.076	0.111	0.214
San Diego, California	San Diego County	0.038	0.074	0.110	0.063	0.054	0.058	0.138	0.215	0.109
San Francisco, California	San Francisco County	0.069	0.179	0.081	0.057	0.031	0.050	0.110	0.122	0.130

*MIC testing range was 0.001–16 µg/mL in 2005–2006; range was 0.008–16 µg/mL during 2007–2013.

Technical Appendix Table 5. Outpatient oral cephalosporin* prescriptions per 1,000 men 10–59 y by county, Gonococcal Isolate Surveillance Project (GISP), 2005–2013

County (and associated GISP site)	2005	2006	2007	2008	2009	2010	2011	2012	2013
Northeast									
Philadelphia, Pennsylvania	70	62	61	59	57	54	76	75	78
Midwest									
Cook County, Illinois (Chicago)	18	20	33	57	56	53	67	67	67
Cuyahoga County, Ohio (Cleveland)	56	75	80	91	81	66	88	86	102
Hennepin County, Minnesota (Minnesota)	21	31	41	64	65	66	76	74	76
South									
Baltimore County, Maryland (Baltimore)	71	64	60	61	60	57	67	68	65
Dallas County, Texas (Dallas)	50	56	67	85	86	86	90	96	91
Fulton County, Georgia (Atlanta)	62	97	93	91	96	96	92	92	93
Guilford County, North Carolina (Greensboro)	78	80	75	75	72	69	75	77	75
Jefferson County, Alabama (Birmingham)	116	137	138	149	140	133	164	162	160
Miami-Dade County, Florida (Miami)	25	27	32	45	44	41	45	45	44
Oklahoma County, Oklahoma (Oklahoma City)	50	60	83	134	127	126	140	144	148
Orleans Parish, Louisiana (New Orleans)	13	39	48	69	66	59	91	84	80
West									
Bernalillo County, New Mexico (Albuquerque)	13	19	36	75	71	73	80	76	74
Clark County, Nevada (Las Vegas)	48	45	52	71	66	64	63	61	62
Denver County, Colorado (Denver)	16	27	32	45	44	42	57	57	55
Honolulu County, Hawaii (Honolulu)	65	64	63	59	55	48	56	56	52
King County, Washington (Seattle)	45	48	50	60	58	53	65	62	59
Los Angeles County, California (Los Angeles)	61	56	54	55	54	50	56	54	54
Maricopa County, Arizona (Phoenix)	33	33	44	74	75	76	81	79	77
Multnomah County, Oregon (Portland)	21	30	32	41	39	38	52	47	47
Orange County, California	62	64	64	67	64	60	65	61	63
San Diego County, California (San Diego)	44	43	40	40	38	38	42	43	43
San Francisco County, California (San Francisco)	7	17	26	42	40	41	58	54	54

*Includes cefaclor, cefadroxil, cefdinir, cefditoren pivoxil, cefixime, cefpodoxime proxetil, cefprozil, ceftibuten, cefuroxime axetil, cephalexin, cephradine, and loracarbef

Technical Appendix Table 6. Outpatient oral macrolide* prescriptions per 1,000 men 10–59 y by county, Gonococcal Isolate Surveillance Project (GISP), 2005–2013

County (and associated GISP site)	2005	2006	2007	2008	2009	2010	2011	2012	2013
Northeast									
Philadelphia, Pennsylvania	103	91	101	112	117	103	139	135	126
Midwest									
Cook County, Illinois (Chicago)	31	32	62	120	130	116	150	145	127
Cuyahoga County, Ohio (Cleveland)	92	108	122	155	155	141	187	169	144
Hennepin County, Minnesota (Minnesota)	34	44	64	113	112	115	132	124	108
South									
Baltimore County, Maryland (Baltimore)	96	88	97	121	125	113	135	133	115
Dallas County, Texas (Dallas)	61	66	98	135	137	131	141	151	142
Fulton County, Georgia (Atlanta)	98	124	134	165	172	155	143	148	133
Guilford County, North Carolina (Greensboro)	109	108	121	140	135	120	132	144	124
Jefferson County, Alabama (Birmingham)	136	159	186	244	261	240	260	280	260
Miami-Dade County, Florida (Miami)	41	45	61	98	112	98	106	110	100
Oklahoma County, Oklahoma (Oklahoma City)	57	62	118	216	204	193	213	217	188
Orleans Parish, Louisiana (New Orleans)	17	62	80	136	135	125	176	165	147
West									
Bernalillo County, New Mexico (Albuquerque)	19	27	53	121	118	120	133	122	111
Clark County, Nevada (Las Vegas)	61	52	65	113	117	107	118	111	105
Denver County, Colorado (Denver)	26	39	49	75	72	66	91	87	81
Honolulu County, Hawaii (Honolulu)	17	26	45	85	88	90	105	92	90
King County, Washington (Seattle)	68	66	77	98	94	81	114	105	88
Los Angeles County, California (Los Angeles)	74	65	70	84	91	85	102	89	89
Maricopa County, Arizona (Phoenix)	49	44	66	123	129	126	144	134	120
Multnomah County, Oregon (Portland)	28	36	45	64	61	60	86	74	65
Orange County, California	107	100	106	125	128	120	136	115	115
San Diego County, California (San Diego)	61	57	57	63	64	67	77	67	66
San Francisco County, California (San Francisco)	17	26	45	85	88	90	105	92	90

*Includes azithromycin, clarithromycin, and erythromycin

Technical Appendix Table 7. Outpatient oral fluoroquinolone* prescriptions per 1,000 men 10–59 y by county, Gonococcal Isolate Surveillance Project (GISP), 2005–2013

County (and associated GISP site)	2005	2006	2007	2008	2009	2010	2011	2012	2013
Northeast									
Philadelphia, Pennsylvania	68	72	75	77	70	62	88	88	89
Midwest									
Cook County, Illinois (Chicago)	19	22	39	70	62	55	71	71	70
Cuyahoga County, Ohio (Cleveland)	47	66	75	87	78	62	86	82	92
Hennepin County, Minnesota (Minneapolis)	17	27	39	65	59	55	64	61	58
South									
Baltimore County, Maryland (Baltimore)	78	71	72	76	65	58	71	70	66
Dallas County, Texas (Dallas)	41	50	68	91	82	74	80	88	87
Fulton County, Georgia (Atlanta)	74	115	119	131	117	108	109	107	104
Guilford County, North Carolina (Greensboro)	70	78	79	84	75	69	77	80	77
Jefferson County, Alabama (Birmingham)	75	103	119	141	130	120	153	180	179
Miami-Dade County, Florida (Miami)	29	33	45	65	61	56	63	63	62
Oklahoma County, Oklahoma (Oklahoma City)	34	46	74	124	108	103	115	120	117
Orleans Parish, Louisiana (New Orleans)	11	45	61	83	72	64	104	107	105
West									
Bernalillo County, New Mexico (Albuquerque)	8	13	23	47	43	43	52	50	46
Clark County, Nevada (Las Vegas)	37	38	48	71	61	54	54	55	57
Denver County, Colorado (Denver)	15	28	35	49	44	39	54	50	47
Honolulu County, Hawaii (Honolulu)	41	47	52	52	48	42	53	53	50
King County, Washington (Seattle)	36	38	44	51	45	39	48	46	41
Los Angeles County, California (Los Angeles)	38	39	42	46	41	37	45	44	44
Maricopa County, Arizona (Phoenix)	22	25	36	63	58	56	59	57	53
Multnomah County, Oregon (Portland)	17	29	33	43	37	37	50	46	44
Orange County, California	49	53	58	62	56	50	53	52	52
San Diego County, California (San Diego)	32	33	33	33	30	29	33	33	33
San Francisco County, California (San Francisco)	7	18	34	60	53	55	74	68	67

*Includes ciprofloxacin, gemifloxacin, levofloxacin,moxifloxacin, norfloxacin, ofloxacin, and trovafloxacin

Technical Appendix Table 8. *Neisseria gonorrhoeae* antimicrobial drug susceptibility (median annual geometric mean MICs and median annual prescribing rates of counties corresponding to the 23 sites continuously participating in the Gonococcal Isolate Surveillance Project (GISP) by site, 2005–2013*

County (associated GISP site)	Total no. isolates	Mean age of men who submitted isolates (SD)	% of isolates from MSM	% of isolates from black men	Antimicrobial drug susceptibility (Median and interdecile range of annual geometric mean MIC values)								Outpatient prescriptions per 1,000 men 10–59 (Median and interdecile range of annual values)					
					Azithromycin		Ciprofloxacin		Cefixime		Ceftriaxone		Macrolide		Fluoroquinolone		Cephalosporin	
					Median	Inter-decile range	Median	Inter-decile range	Median	Inter-decile range	Median	Inter-decile range	Median	Inter-decile range	Median	Inter-decile range	Median	Inter-decile range
Northeast																		
Philadelphia, Pennsylvania	2486	31 (10)	21	88	0.298	0.143	0.038	0.048	0.02	0.012	0.01	0.005	112	48	75	27	62	24
Midwest																		
Cook County (Chicago), Illinois	2290	28 (9)	26	82	0.269	0.098	0.021	0.044	0.019	0.014	0.01	0.007	120	119	62	52	56	49
Cuyahoga County (Cleveland), Ohio	2081	28 (9)	8	94	0.258	0.106	0.016	0.01	0.017	0.011	0.009	0.006	144	95	78	45	81	46
Hennepin County (Minneapolis), Minnesota	1305	30 (9)	31	70	0.314	0.143	0.03	0.068	0.022	0.017	0.012	0.01	112	98	58	48	65	55
South																		
Baltimore County (Baltimore), Maryland	2552	28 (9)	11	97	0.234	0.125	0.016	0.046	0.019	0.015	0.009	0.006	115	47	71	20	64	14
Dallas County (Dallas), Texas	2576	26 (8)	16	100	0.1	0.094	0.022	0.026	0.019	0.014	0.009	0.006	135	90	80	50	86	46
Fulton County (Atlanta), Georgia	2068	28 (9)	19	99	0.118	0.268	0.02	0.024	0.016	0.007	0.009	0.005	143	74	109	57	93	35
Guilford County (Greensboro), North Carolina	1497	27 (9)	9	94	0.15	0.156	0.017	0.018	0.016	0.012	0.009	0.006	124	36	77	15	75	11
Jefferson County (Birmingham), Alabama	2004	27 (9)	4	97	0.228	0.168	0.029	0.052	0.019	0.012	0.009	0.006	244	144	130	105	140	48
Miami-Dade County (Miami), Florida	1916	28 (10)	22	88	0.166	0.145	0.035	0.053	0.018	0.008	0.01	0.094	98	71	61	36	44	20
Oklahoma County (Oklahoma City), Oklahoma	2181	27 (9)	6	82	0.222	0.128	0.015	0.017	0.017	0.012	0.009	0.006	193	160	108	90	127	98
Orleans Parish (New Orleans), Louisiana	1703	27 (9)	11	95	0.139	0.143	0.029	0.026	0.017	0.007	0.009	0.004	135	159	72	96	66	78
West																		
Bernalillo County (Albuquerque), New Mexico	1112	28 (9)	24	19	0.136	0.168	0.026	0.057	0.018	0.008	0.01	0.006	118	114	43	44	73	67
Clark County (Las Vegas), Nevada	2617	28 (9)	21	63	0.259	0.125	0.029	0.065	0.019	0.015	0.01	0.008	107	66	54	34	62	26
Denver County (Denver), Colorado	2069	29 (9)	32	50	0.161	0.158	0.033	0.036	0.02	0.012	0.01	0.007	72	65	44	39	44	41
Honolulu County (Honolulu), Hawaii	705	31 (9)	47	41	0.152	0.11	0.076	0.251	0.02	0.023	0.01	0.008	88	88	50	12	56	17

County (associated GISP site)	Total no. isolates	Mean age of men who submitted isolates (SD)	% of isolates from MSM	% of isolates from black men	Antimicrobial drug susceptibility (Median and interdecile range of annual geometric mean MIC values)								Outpatient prescriptions per 1,000 men 10–59 (Median and interdecile range of annual values)					
					Azithromycin		Ciprofloxacin		Cefixime		Ceftriaxone		Macrolide		Fluoroquinolone		Cephalosporin	
					Median	Inter-decile range	Median	Inter-decile range	Median	Inter-decile range	Median	Inter-decile range	Median	Inter-decile range	Median	Inter-decile range	Median	Inter-decile range
King County (Seattle), Washington	1682	34 (10)	62	34	0.158	0.092	0.123	0.488	0.03	0.024	0.011	0.01	88	48	44	15	58	20
Los Angeles County (Los Angeles), California	1384	29 (9)	53	52	0.195	0.12	0.041	0.104	0.022	0.015	0.01	0.007	85	37	42	9	54	11
Maricopa County (Phoenix), Arizona	2382	29 (9)	29	36	0.295	0.201	0.028	0.029	0.019	0.01	0.01	0.005	123	100	56	41	75	48
Multnomah County (Portland), Oregon	1318	31 (10)	56	30	0.217	0.11	0.06	0.4	0.022	0.015	0.012	0.01	61	58	37	33	39	31
Orange County, California	984	31 (10)	58	100	0.28	0.29	0.076	0.169	0.024	0.016	0.011	0.004	115	36	53	13	64	7
San Diego County (San Diego), California	1869	34 (10)	74	22	0.292	0.25	0.074	0.177	0.028	0.015	0.012	0.005	64	20	33	4	42	6
San Francisco County (San Francisco), California	2294	35 (10)	71	29	0.204	0.118	0.081	0.148	0.023	0.016	0.01	0.006	88	88	55	67	41	51

*MSM, men who have sex with men.
